# Methyl 3-hydr­oxy-4-(3-methyl­but-2-en­yloxy)benzoate

**DOI:** 10.1107/S160053680900806X

**Published:** 2009-03-11

**Authors:** Mei-yan Wei, Zhen Liu, Xiu-li Zhang, Chang-lun Shao, Chang-yun Wang

**Affiliations:** a School of Pharmacy, Guangdong Medical College, Dongguan, Guangdong 523808, People’s Republic of China; b College of Chemistry and Chemical Engineering, Luoyang Normal University, Luoyang, Henan 471022, People’s Republic of China; c School of Medicine and Pharmacy, Ocean University of China, Qingdao, Shandong 266003, People’s Republic of China

## Abstract

The title compound, C_13_H_16_O_4_, was isolated from culture extracts of the endophytic fungus *Cephalosporium* sp. The ester and ether substituents are twisted only slightly out of the benzene ring plane, making dihedral angles of 2.16 (2) and 3.63 (5)°, respectively. The non-H atoms of all three substituents are almost coplanar with the benzene ring, with an r.m.s. deviation of 0.0284 Å from the mean plane through all non-H atoms in the structure. A weak intra­molecular O—H⋯O hydrogen bond contributes to this conformation. In the crystal structure, mol­ecules are linked into a one-dimensional chain by inter­molecular O—H⋯O hydrogen bonds. Weak non-classical C—H⋯π contacts are also observed in the structure.

## Related literature

For structures with C—H⋯O and C—H⋯π contacts, see: Nangia (2002 ▶); Umezawa *et al.* (1999 ▶). For new bioactive secondary metabolites from the endophytic strain B60, see: Shao *et al.* (2007 ▶, 2008 ▶). For an investigation of the endophytic fungus, see: Shao *et al.* (2008 ▶). For a related structure, see: Huang *et al.* (2005 ▶).
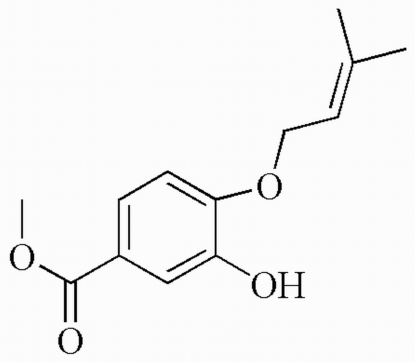


## Experimental

### 

#### Crystal data

C_13_H_16_O_4_
*M* *_r_* = 236.26
Triclinic, 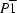

*a* = 7.8401 (16) Å
*b* = 8.3899 (17) Å
*c* = 11.099 (2) Å
α = 100.655 (3)°
β = 98.771 (3)°
γ = 115.456 (3)°
*V* = 625.3 (2) Å^3^
*Z* = 2
Mo *K*α radiation
μ = 0.09 mm^−1^
*T* = 291 K
0.40 × 0.38 × 0.35 mm

#### Data collection

Bruker APEXII CCD diffractometer
Absorption correction: multi-scan (*SADABS*; Sheldrick, 1996 ▶) *T*_min_ = 0.964, *T*_max_ = 0.968
4817 measured reflections
2420 independent reflections
2058 reflections with *I* > 2σ(*I*)
*R*_int_ = 0.014

#### Refinement

*R*[*F* ^2^ > 2σ(*F* ^2^)] = 0.051
*wR*(*F* ^2^) = 0.161
*S* = 1.05
2420 reflections
158 parameters
H atoms treated by a mixture of independent and constrained refinement
Δρ_max_ = 0.23 e Å^−3^
Δρ_min_ = −0.25 e Å^−3^

### 

Data collection: *APEX2* (Bruker, 2004 ▶); cell refinement: *SAINT* (Bruker, 2004 ▶); data reduction: *SAINT*; program(s) used to solve structure: *SHELXS97* (Sheldrick, 2008 ▶); program(s) used to refine structure: *SHELXL97* (Sheldrick, 2008 ▶); molecular graphics: *SHELXTL* (Sheldrick, 2008 ▶) and *DIAMOND* (Brandenburg, 2006 ▶); software used to prepare material for publication: *SHELXTL* and *PLATON* (Spek, 2009 ▶).

## Supplementary Material

Crystal structure: contains datablocks global, I. DOI: 10.1107/S160053680900806X/sj2576sup1.cif

Structure factors: contains datablocks I. DOI: 10.1107/S160053680900806X/sj2576Isup2.hkl

Additional supplementary materials:  crystallographic information; 3D view; checkCIF report

## Figures and Tables

**Table 1:** Hydrogen-bond geometry (Å, °)

*D*—H⋯*A*	*D*—H	H⋯*A*	*D*⋯*A*	*D*—H⋯*A*
O3—H3*A*⋯O4	0.84 (3)	2.16 (2)	2.6519 (15)	117 (2)
O3—H3*A*⋯O2^i^	0.84 (3)	2.20 (3)	2.9111 (16)	143 (2)
C9—H9*B*⋯*Cg*1^ii^	0.97	2.90	3.7483 (2)	146
C13—H13*B*⋯*Cg*1^iii^	0.96	2.96	3.688 (3)	134

Symmetry codes: (i) 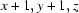; (ii) 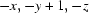; (iii) 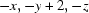. *Cg*1 is the centroid of the C3–C8 ring.

**Fig. 1.**
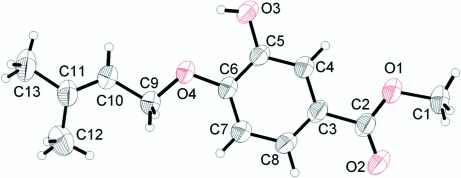
The structure of (I) with displacement ellipsoids for the non-hydrogen atoms drawn at the 30% probability level.

**Fig. 2.**
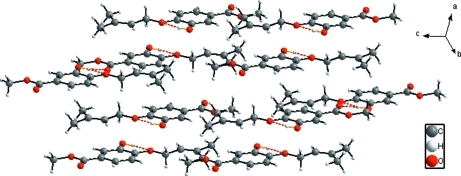
Crystal packing of (I) viewed down the b axis with hydrogen bonds drawn as dashed lines.

## supplementary crystallographic information

### Comment

Marine fungi have proven to be a rich source of novel structural compounds with interesting biological activities and a high level of biodiversity. As a continuation of our previous investigations aimed at finding new bioactive compounds, we found that an unidentified endophytic strain B60 isolated from the mangrove tree can produce new metabolites (Shao *et al.* 2007; Shao *et al.* 2008).

Although the structure of the title compound was previously elucidated on the basis of spectroscopic analysis (Shao *et al.* 2008), we have now determined its solid state structure, Fig. 1, which is reported here. All bond lengths and angles in the molecule are in good agreement with those reported in a related  structure by  Huang *et al.* (2005). In the title compound, the most striking feature is the interesting arrangement of the molecules, which linked to form a one-dimensional chain by intermolecular O—H···O hydrogen bonds, Table 1, Fig. 2.  Further, weak non-classical C—H···π contacts, similar to those previously reported (Nangia, 2002; Umezawa *et al.* 1999) are also observed, in which C9—H9B and C13—H13B act as donors with the benzene ring as the acceptor.

### Experimental

An unidentified fungus (No. B60) was deposited in the School of Chemistry and Chemical Engineering, Sun Yat-sen University, Guangzhou, People's Republic of China. Strain No. B60 was cultivated without shaking in GYT medium (10 g of glucose, 2 g of peptone /*L*, 1 g of yeast extract /*L*, 2.5 g of NaCl, 1*L* of water) at 298 K for 4 weeks. The cultures (120 *L*) were filtered through cheesecloth. The filtrate was concentrated to 3 *L* below 323 K, then extracted five times by shaking with an equal volume of ethyl acetate. The extract was evaporated under reduced pressure below 323 K. The combined organic extracts were subjected to silica-gel column chromatography, eluting with petroleum ether/ethyl acetate (9:1, v:v), to yield the title compound, which was confirmed by spectral data including NMR and EI—MS. Crystals of the title compound were obtained by evaporation of an ethyl acetate solution.

### Refinement

All H atoms were positioned geometrically and treated as riding, with C—H bonding lengths constrained to 0.93 (aromatic CH), 0.96 Å (methyl CH_3_), 0.97 Å (methylene CH_2_), and O—H = 0.84 Å, and with *U*ĩso~(H) = 1.2Ueq(CH) or *U*ĩso~(H) = 1.5Ueq(CH_3_, methylene C or OH).

### Crystal data

**Table:**

C_13_H_16_O_4_	*Z* = 2
*M**_r_* = 236.26	*F*(000) = 252
Triclinic, *P*1	*D*_x_ = 1.255 Mg m^−^^3^
Hall symbol: -P 1	Mo *K*α radiation, λ = 0.71073 Å
*a* = 7.8401 (16) Å	Cell parameters from 4817 reflections
*b* = 8.3899 (17) Å	θ = 1.9–26.0°
*c* = 11.099 (2) Å	µ = 0.09 mm^−^^1^
α = 100.655 (3)°	*T* = 291 K
β = 98.771 (3)°	Block, colorless
γ = 115.456 (3)°	0.40 × 0.38 × 0.35 mm
*V* = 625.3 (2) Å^3^	

### Data collection

**Table:**

Bruker APEXII CCD diffractometer	2420 independent reflections
Radiation source: fine-focus sealed tube	2058 reflections with *I* > 2σ(*I*)
graphite	*R*_int_ = 0.014
φ and ω scans	θ_max_ = 26.0°, θ_min_ = 1.9°
Absorption correction: multi-scan (*SADABS*; Sheldrick, 1996)	*h* = −9→9
*T*_min_ = 0.964, *T*_max_ = 0.968	*k* = −10→10
4817 measured reflections	*l* = −13→13

### Refinement

**Table:**

Refinement on *F*^2^	Primary atom site location: structure-invariant direct methods
Least-squares matrix: full	Secondary atom site location: difference Fourier map
*R*[*F*^2^ > 2σ(*F*^2^)] = 0.051	Hydrogen site location: inferred from neighbouring sites
*wR*(*F*^2^) = 0.161	H atoms treated by a mixture of independent and constrained refinement
*S* = 1.05	*w* = 1/[σ^2^(*F*_o_^2^) + (0.1024*P*)^2^ + 0.0667*P*] where *P* = (*F*_o_^2^ + 2*F*_c_^2^)/3
2420 reflections	(Δ/σ)_max_ < 0.001
158 parameters	Δρ_max_ = 0.23 e Å^−^^3^
0 restraints	Δρ_min_ = −0.25 e Å^−^^3^

### Special details

**Table:**

Geometry. All e.s.d.'s (except the e.s.d. in the dihedral angle between two l.s. planes) are estimated using the full covariance matrix. The cell e.s.d.'s are taken into account individually in the estimation of e.s.d.'s in distances, angles and torsion angles; correlations between e.s.d.'s in cell parameters are only used when they are defined by crystal symmetry. An approximate (isotropic) treatment of cell e.s.d.'s is used for estimating e.s.d.'s involving l.s. planes.
Refinement. Refinement of *F*^2^ against ALL reflections. The weighted *R*-factor *wR* and goodness of fit *S* are based on *F*^2^, conventional *R*-factors *R* are based on *F*, with *F* set to zero for negative *F*^2^. The threshold expression of *F*^2^ > σ(*F*^2^) is used only for calculating *R*-factors(gt) *etc*. and is not relevant to the choice of reflections for refinement. *R*-factors based on *F*^2^ are statistically about twice as large as those based on *F*, and *R*- factors based on ALL data will be even larger.

### Fractional atomic coordinates and isotropic or equivalent isotropic displacement parameters (Å^2^)

**Table:**

	*x*	*y*	*z*	*U*_iso_*/*U*_eq_	
C1	−0.4408 (3)	0.2951 (3)	0.50642 (16)	0.0716 (5)	
H1A	−0.3697	0.3458	0.5944	0.107*	
H1B	−0.4681	0.1692	0.4784	0.107*	
H1C	−0.5616	0.3009	0.4960	0.107*	
C2	−0.4029 (2)	0.34671 (19)	0.30738 (14)	0.0490 (4)	
C3	−0.2748 (2)	0.46837 (17)	0.24198 (13)	0.0453 (3)	
C4	−0.0924 (2)	0.61796 (19)	0.30835 (13)	0.0481 (4)	
H4A	−0.0474	0.6410	0.3955	0.058*	
C5	0.02077 (19)	0.73115 (19)	0.24510 (13)	0.0457 (3)	
C6	−0.04479 (19)	0.69490 (18)	0.11391 (13)	0.0452 (3)	
C7	−0.2253 (2)	0.5465 (2)	0.04830 (14)	0.0558 (4)	
H7A	−0.2702	0.5224	−0.0389	0.067*	
C8	−0.3388 (2)	0.4343 (2)	0.11268 (14)	0.0544 (4)	
H8A	−0.4597	0.3346	0.0682	0.065*	
C9	0.0269 (2)	0.7902 (2)	−0.07071 (14)	0.0557 (4)	
H9A	−0.0895	0.8036	−0.0942	0.067*	
H9B	0.0003	0.6684	−0.1169	0.067*	
C10	0.1952 (2)	0.9336 (2)	−0.10072 (15)	0.0578 (4)	
H10A	0.3061	1.0114	−0.0342	0.069*	
C11	0.1993 (2)	0.9589 (2)	−0.21421 (14)	0.0546 (4)	
C12	0.0315 (3)	0.8410 (3)	−0.32730 (17)	0.0836 (6)	
H12A	−0.0725	0.7516	−0.3026	0.125*	
H12B	0.0724	0.7793	−0.3892	0.125*	
H12C	−0.0138	0.9160	−0.3632	0.125*	
C13	0.3751 (3)	1.1067 (3)	−0.2368 (2)	0.0752 (5)	
H13A	0.4748	1.1741	−0.1582	0.113*	
H13B	0.3387	1.1889	−0.2699	0.113*	
H13C	0.4242	1.0518	−0.2968	0.113*	
O1	−0.32509 (16)	0.39913 (15)	0.43211 (10)	0.0640 (4)	
O2	−0.56125 (16)	0.21602 (16)	0.25485 (12)	0.0702 (4)	
O3	0.19557 (16)	0.87793 (16)	0.31218 (11)	0.0663 (4)	
H3A	0.231 (4)	0.943 (4)	0.263 (2)	0.102 (8)*	
O4	0.08089 (14)	0.81567 (13)	0.06303 (9)	0.0537 (3)	

### Atomic displacement parameters (Å^2^)

**Table:**

	*U*^11^	*U*^22^	*U*^33^	*U*^12^	*U*^13^	*U*^23^
C1	0.0731 (11)	0.0727 (11)	0.0615 (10)	0.0165 (9)	0.0292 (9)	0.0378 (9)
C2	0.0446 (7)	0.0438 (7)	0.0559 (8)	0.0141 (6)	0.0170 (6)	0.0222 (6)
C3	0.0414 (7)	0.0383 (7)	0.0526 (8)	0.0122 (6)	0.0151 (6)	0.0191 (6)
C4	0.0452 (7)	0.0459 (7)	0.0446 (7)	0.0117 (6)	0.0118 (6)	0.0186 (6)
C5	0.0377 (7)	0.0407 (7)	0.0492 (7)	0.0089 (5)	0.0108 (5)	0.0166 (6)
C6	0.0434 (7)	0.0397 (7)	0.0494 (8)	0.0127 (6)	0.0169 (6)	0.0192 (6)
C7	0.0535 (8)	0.0494 (8)	0.0451 (7)	0.0067 (7)	0.0093 (6)	0.0177 (6)
C8	0.0456 (7)	0.0441 (7)	0.0523 (8)	0.0029 (6)	0.0078 (6)	0.0166 (6)
C9	0.0579 (9)	0.0501 (8)	0.0481 (8)	0.0119 (7)	0.0171 (6)	0.0208 (6)
C10	0.0588 (9)	0.0500 (8)	0.0534 (8)	0.0114 (7)	0.0210 (7)	0.0205 (7)
C11	0.0688 (10)	0.0527 (8)	0.0556 (8)	0.0316 (8)	0.0311 (7)	0.0252 (7)
C12	0.0918 (14)	0.0981 (15)	0.0565 (10)	0.0370 (12)	0.0193 (10)	0.0314 (10)
C13	0.0933 (14)	0.0714 (11)	0.0806 (12)	0.0382 (11)	0.0533 (11)	0.0417 (10)
O1	0.0574 (6)	0.0626 (7)	0.0561 (6)	0.0075 (5)	0.0189 (5)	0.0305 (5)
O2	0.0530 (7)	0.0590 (7)	0.0689 (7)	−0.0030 (5)	0.0148 (5)	0.0275 (6)
O3	0.0486 (6)	0.0604 (7)	0.0555 (7)	−0.0055 (5)	0.0056 (5)	0.0255 (5)
O4	0.0496 (6)	0.0489 (6)	0.0470 (6)	0.0055 (5)	0.0153 (4)	0.0212 (5)

### Geometric parameters (Å, °)

**Table:**

C1—O1	1.4418 (17)	C8—H8A	0.9300
C1—H1A	0.9600	C9—O4	1.4289 (18)
C1—H1B	0.9600	C9—C10	1.489 (2)
C1—H1C	0.9600	C9—H9A	0.9700
C2—O2	1.2066 (18)	C9—H9B	0.9700
C2—O1	1.3300 (18)	C10—C11	1.319 (2)
C2—C3	1.4808 (18)	C10—H10A	0.9300
C3—C8	1.378 (2)	C11—C12	1.484 (3)
C3—C4	1.399 (2)	C11—C13	1.500 (2)
C4—C5	1.3763 (18)	C12—H12A	0.9600
C4—H4A	0.9300	C12—H12B	0.9600
C5—O3	1.3594 (17)	C12—H12C	0.9600
C5—C6	1.397 (2)	C13—H13A	0.9600
C6—O4	1.3563 (16)	C13—H13B	0.9600
C6—C7	1.386 (2)	C13—H13C	0.9600
C7—C8	1.382 (2)	O3—H3A	0.84 (3)
C7—H7A	0.9300		
			
O1—C1—H1A	109.5	O4—C9—C10	106.75 (12)
O1—C1—H1B	109.5	O4—C9—H9A	110.4
H1A—C1—H1B	109.5	C10—C9—H9A	110.4
O1—C1—H1C	109.5	O4—C9—H9B	110.4
H1A—C1—H1C	109.5	C10—C9—H9B	110.4
H1B—C1—H1C	109.5	H9A—C9—H9B	108.6
O2—C2—O1	123.26 (13)	C11—C10—C9	125.11 (15)
O2—C2—C3	124.42 (14)	C11—C10—H10A	117.4
O1—C2—C3	112.31 (12)	C9—C10—H10A	117.4
C8—C3—C4	119.39 (13)	C10—C11—C12	121.92 (15)
C8—C3—C2	118.91 (13)	C10—C11—C13	121.91 (16)
C4—C3—C2	121.69 (13)	C12—C11—C13	116.16 (15)
C5—C4—C3	120.19 (13)	C11—C12—H12A	109.5
C5—C4—H4A	119.9	C11—C12—H12B	109.5
C3—C4—H4A	119.9	H12A—C12—H12B	109.5
O3—C5—C4	119.06 (13)	C11—C12—H12C	109.5
O3—C5—C6	120.93 (12)	H12A—C12—H12C	109.5
C4—C5—C6	120.01 (12)	H12B—C12—H12C	109.5
O4—C6—C7	126.11 (13)	C11—C13—H13A	109.5
O4—C6—C5	114.18 (12)	C11—C13—H13B	109.5
C7—C6—C5	119.71 (13)	H13A—C13—H13B	109.5
C8—C7—C6	119.92 (14)	C11—C13—H13C	109.5
C8—C7—H7A	120.0	H13A—C13—H13C	109.5
C6—C7—H7A	120.0	H13B—C13—H13C	109.5
C3—C8—C7	120.78 (13)	C2—O1—C1	117.18 (12)
C3—C8—H8A	119.6	C5—O3—H3A	105.7 (18)
C7—C8—H8A	119.6	C6—O4—C9	118.40 (11)
			
O2—C2—C3—C8	0.9 (2)	C5—C6—C7—C8	−0.6 (2)
O1—C2—C3—C8	−178.13 (12)	C4—C3—C8—C7	−0.3 (2)
O2—C2—C3—C4	−179.95 (14)	C2—C3—C8—C7	178.79 (13)
O1—C2—C3—C4	1.0 (2)	C6—C7—C8—C3	0.2 (3)
C8—C3—C4—C5	0.8 (2)	O4—C9—C10—C11	−177.76 (15)
C2—C3—C4—C5	−178.28 (12)	C9—C10—C11—C12	−0.9 (3)
C3—C4—C5—O3	178.57 (13)	C9—C10—C11—C13	−179.89 (15)
C3—C4—C5—C6	−1.2 (2)	O2—C2—O1—C1	−1.6 (2)
O3—C5—C6—O4	0.8 (2)	C3—C2—O1—C1	177.47 (13)
C4—C5—C6—O4	−179.48 (12)	C7—C6—O4—C9	−0.6 (2)
O3—C5—C6—C7	−178.70 (14)	C5—C6—O4—C9	179.99 (12)
C4—C5—C6—C7	1.0 (2)	C10—C9—O4—C6	−176.59 (12)
O4—C6—C7—C8	−179.96 (13)		

### Hydrogen-bond geometry (Å, °)

**Table:**

*D*—H···*A*	*D*—H	H···*A*	*D*···*A*	*D*—H···*A*
O3—H3A···O4	0.84 (3)	2.16 (2)	2.6519 (15)	117 (2)
O3—H3A···O2^i^	0.84 (3)	2.20 (3)	2.9111 (16)	143 (2)
C9—H9B···Cg1^ii^	0.97	2.90	3.7483 (2)	146
C13—H13B···Cg1^iii^	0.96	2.96	3.688 (3)	134

Symmetry codes: (i) *x*+1, *y*+1, *z*; (ii) −*x*, −*y*+1, −*z*; (iii) −*x*, −*y*+2, −*z*.

## References

[bb1] Brandenburg, K. (2006). *DIAMOND* Crystal Impact GbR, Bonn, Germany.

[bb2] Bruker (2004). *APEX2 *and *SAINT* . Bruker AXS Inc., Madison, Wisconsin, USA.

[bb3] Huang, H.-R., Feng, X.-L., She, Z.-G., Lin, Y.-C., Vrijmoed, L. L. P. & Jones, E. B. G. (2005). *Acta Cryst.* E**61**, o282–o283.

[bb4] Nangia, A. (2002). *CrystEngComm*, **4**, 93–101.

[bb5] Shao, C. L., Guo, Z. Y., Peng, H., Peng, G. T., Huang, Z. J., She, Z. G., Lin, Y. C. & Zhou, S. N. (2007). *Chem. Nat. Compd*, **43**, 377–379.

[bb6] Shao, C. L., Hu, G. P., Zhang, X. L., Wang, C. Y., She, Z. G. & Lin, Y. C. (2008). *Acta Sci. Nat. Univ. Sunyatseni*, **47**, 133–134.

[bb7] Sheldrick, G. M. (1996). *SADABS* University of Göttingen, Germany.

[bb8] Sheldrick, G. M. (2008). *Acta Cryst.* A**64**, 112–122. 10.1107/S0108767307043930 18156677

[bb9] Spek, A. L. (2009). *Acta Cryst.* D**65**, 148–155. 10.1107/S090744490804362X PMC2631630 19171970

[bb10] Umezawa, Y., Tsuboyama, S., Takahashi, H., Uzawa, J. & Nishio, M. (1999). *Bioorg. Med. Chem.* **7**, 2021–2026. 10.1016/s0968-0896(99)00123-6 10530951

